# Individual Differences in Foraging Strategies of Parasitic Sabre-Tooth Blennies

**DOI:** 10.1371/journal.pone.0045998

**Published:** 2012-09-28

**Authors:** Andrea Bshary, Redouan Bshary

**Affiliations:** Université de Neuchâtel, Institut de Biologie, Eco-Ethologie, Neuchâtel, Switzerland; Institut Pluridisciplinaire Hubert Curien, France

## Abstract

Originally, evolutionary game theory typically predicted that optimal behaviour in a given situation is uniform or bimodal. However, the growing evidence that animals behave more variably while individuals may differ consistently in their behaviour, has led to the development of models that predict a distribution of strategies. Here we support the importance of such models in a study on a coral reef fish host–parasite system. Parasitic blennies (*Plagiotremus sp*.) regularly attack other fishes to bite off scales and mucus. Individuals of some victim species react to being bitten with punishing the parasite through aggressive chasing. Our field observations and laboratory experiments show that individual blennies differ markedly in how they incorporate being punished into their foraging decisions. We discuss how these differences may affect the payoff structure and hence the net effect of punishment on punishers and on the appearance of a public good for look-alikes.

## Introduction

Cooperation in groups that consist of more than two unrelated individuals has attracted considerable research interest. A key problem for achieving cooperation in larger groups is typically illustrated with the standard public goods game. In this game each group member may contribute to a communal purse. Contributions create additional value, which in the game is achieved by the experimenter matching contributions. After each round the group’s gains are equally split between group members, irrespective of how much each individual contributed. These rules typically lead to a situation where it is in the group interest that everybody contributes while for individuals contributions are altruistic in the evolutionary sense [1], i.e. contributions reduce the actor’s direct benefits. It has been repeatedly demonstrated that humans fail to cooperate in this game [2], [3], which fits the ‘tragedy of the commons’ idea developed by Hardin 1968.

However, variants of the public goods game may allow cooperative solutions. For example, individuals may benefit from contributing to a public good if this raises their image score and hence increases the probability of receiving help in other contexts [2]. Also, if human players have the option to punish free–riders (non contributing individuals) then stable cooperation may be achieved [3], even though large cultural differences exist [4]–[6]. Most importantly, it has been argued that cooperative behaviour in n–player interactions may readily emerge if some assumptions of the standard public goods game are relaxed. For example, the assumption of a linear relationship between the amount of contribution and the size of the public good is arguably rarely fulfilled in reality [7]. If one assumes a sigmoid or step function to describe the relationship between the number of contributors and the size of the public good, contributions by few individuals may yield high group productivity that cannot be much enhanced by further contributions [7]. Under these circumstances, contributions are no longer altruistic but contributions and free–riding are under negative frequency dependent selection [7]–[10] as captured in the so-called volunteer’s dilemma game [11]: as long as not enough volunteers have been recruited an individual’s best response is to contribute; while if there are enough volunteers to produce the public good an individual’s best option is to free-ride. Stable contributions to a public good may also emerge if individuals gain disproportionally from their own contributions [11]. For example, individual bacteria gain disproportionally from their own extra–cellular compounds due to their spatial proximity (examples in [12]).

Recently, the idea that individuals gaining disproportionate benefits from their own contributions leads to stable contributions to public goods has been applied to a host–parasite system involving reef fishes and parasitic scale eating blennies of the genus *Plagiotremus*. Reef fish regularly get attacked by parasitic blennies (*Plagiotremus sp*) and many species react to this by chasing the parasite [13]. This chasing functions as punishment sensu Clutton–Brock & Parker [14] as the momentary costs yield future benefits: chasing decreases the probability that the chasing individual gets attacked in the future [15]. In shoaling species this self–serving punishment can additionally create a public good, as the parasites are more likely to avoid members of the group for the next attack if they were chased by one individual [15]. As the probability of punishment correlates slightly negatively with group size [13] the relation between group size and public good seems to follow a non-linear benefit function [7], [8], [16].

The emergence of a public good due to self–serving contributions may depend critically on the foraging decision rules of the blenny. While Bshary & Bshary [15] found a significantly increased probability that blennies switch victim species in response to individual punishment, there appeared to be quite some variance in the field data. In the past such variance was often ignored as game theoretic models typically predicted uniform or bimodal evolutionarily stable strategies [17]. However, there is a growing literature that demonstrates considerable and often persistent variation between individuals [18], [19], and theoretical models demonstrate that such variation may be adaptive [20]–[22]. Most examples are on animal personality but of particular interest to our study are observations that individual predators or ectoparasites of one species may specialize on different victim species and/or hunting strategies. For example, individual leopards may specialise on either antelopes or monkeys [23], and individual scale eating cichlids specialize on attacking their victims from either the left or the right side [24], [25].

The aim of the current paper was to study the foraging decision rules of sabre–tooth blennies with a special emphasis of the question whether there is individual variation in blenny foraging strategies. We were particularly interested in two parameters: the importance of location for an attack and the probability of switching between victim species as a function of victim response. Location of attack is potentially important because groups of victims may have a spatial structure [26] and use a preferred microhabitat compared to other victim species [27]. Therefore, if a blenny changes location in response to punishment it will be more likely to attack another individual rather than the punisher AND more likely an individual of another species. On the other hand, if a blenny remains put and just avoids the punisher the blenny might be more likely to bite a conspecific.

In a first step we reanalyzed the data of Bshary & Bshary [15] to test whether individual blennies differ significantly with respect to the probability that they switch victim species after being aggressed in interactions with a highly abundant victim species, the females of scalefin anthias, *Pseudanthias squamipinnis*. We further analysed whether the probability of switching after punishment correlates with the importance female anthias have in the blenny’s diet and/or with the probability that female anthias punish.

As a second step we performed experiments in the laboratory to investigate the foraging decision rules of individual blennies. We offered blennies simultaneously two small plates covered with mashed prawn. The plates could look quite similar or very different. In the first two experiments neither plate would respond to a blenny taking a bite with aggression (‘no punishment’) which allowed us to explore spontaneous preferences for a location and/or an individual in attacks and whether the blennies need to keep an individual in sight in order to be able to attack it repeatedly:

- If each plate remains in the same spot and in sight, will blennies bite at random or will they focus on one particular victim?
- If plate positions are counterbalanced and plates are out of sight between trials, will blennies bite at random or will they focus on one particular victim or will they focus on one location?

In a third experiment we confronted blennies with three plates that were presented pair–wise in all possible combinations and in randomized positions. Two plates looked very similar to each other while the third plate looked very different. All three plates punished with 50% probability. In such a set–up there is nothing to learn for a blenny. We asked whether blennies would nevertheless show spontaneous adjustment in current choices based on their experience in the previous trial. More specifically we asked whether punishment would affect the likelihood to switch to another location, to another individual/plate or to refrain from taking a bite. Our observations in the field suggested that punishment can have different effects in different individuals.

In all experiments we asked whether blennies show general decision rules or whether individuals differed significantly in their decisions. We will discuss how our findings relate to the idea that punishment of blennies by victims constitutes a self–serving contribution to a public good in a shoaling reef fish species.

## Methods

### Ethics Statement

This study was carried out in strict accordance with the ethical guidelines for research on vertebrates. Ethical permission for the laboratory experiments were obtained from the ethical committee of the University of Queensland, permit number SBS/189/09. Field observations at Ras Mohammed National Park were conducted with permission from the Egyptian Environmental Affairs Agency (EEAA) in Cairo. The EEAA does not provide permit numbers but the local Park rangers ensure that only approved projects take place.

### A) Field Data

#### Study site

Field data were collected in May 2005, May 2006 and June 2007 in the Red Sea, at Ras Mohammed National Park in Sinai, Egypt. The study site was at Mersa Bareika (27°47′20.5′′ N, 34°13′28.7′′ E). In this area, incoming sand through wadis led to the formation of patch reefs which are separated from each other by sand. Observations took place at 20 small reef patches (estimated size between 3.5 m^3^ and 30 m^3^) located in shallow water (bottom depth between 1.5 and 6 m).

#### Study species

The two blenny species studied, *Plagiotremus rhinorhynchus* and *P. tapeinosoma*, occur in the tropical Indo–West and Central Pacific and occupy small territories. Both blenny species are lepidophagous (scale eating) parasites that attack other fish to forage. Usually they sneak up on their victims from behind and bite off small chunks of skin, mucus and scales [28], [29], [15].

For the victims we focussed on female scalefin anthias, *Pseudanthias squamipinnis*, which is a sexually dimorphic, protogynous reef fish occurring in the Indo Pacific in groups of up to several thousand individuals. It is one of the most abundant species on our study reef patches. There is a spatial structure within the anthias at these patches: individuals differ with respect to the preferential use of certain areas [26]. Females are more numerous than males and they are common victims of the two blenny species we observed. Anthias individuals at one patch are not more closely related with each other than with individuals from neighbouring patches [30]. This excludes the possibility that any cooperation we observed is due to kin selection.

#### Data collection

Observations were carried out using scuba equipment and sitting on the surrounding sand 2–3 m in front of the reef patch. One observation session lasted 60 minutes. In total 20 blennies were observed. In 2005, we studied one blenny in detail for 16 hours. In 2006, we studied eleven blennies for 2–4 hours each, while in 2007 eight blennies were observed for 5–8 hours each. Variation in observation duration was due to blennies disappearing and/or spending much time in their hiding holes during single sessions. A total of 95 hours of observations were recorded. We choose reef patches that showed a high abundance of *P. squamipinnis* females (between 60 and 350 individuals) to get a large sample size of interactions with this species.

All interactions between the blenny and another fish were continuously observed over the entire observation period, and, immediately after the observation, the following data was noted on a Plexiglas plate:

1. Category of victim species: *Pseudanthias squamipinnis* female or other
2. Type of interaction between blenny and female anthias victims:
  - unprovoked aggression by the “victim”
  - biting attempt followed by a non–aggressive response of the victim
  - biting attempt followed by aggressive response of the victim
  - bite followed by a non–aggressive response of the victim
  - bite followed by aggressive response of the victim

We scored an ‘aggression’ when a victim swam quickly toward a blenny and consequently, the blenny swam away. We scored ‘unprovoked aggression’ if a fish chased a blenny passing in front and hence not obviously intending to attack the chaser. Successful biting attempts are typically proceeded by the blenny approaching a fish from behind in a characteristic ‘stop and go’ manner [28], [29]. We scored any observed stop and go approach without an actual bite as a biting attempt. We scored ‘aggressive response’ if the potential or real victim turned round and swam towards the blenny. A ‘non–aggressive response’ was scored if the potential or real victim swam away from the blenny or did not move at all.

#### Data analysis

From the sequence of interactions between the blennies and their victim species we extracted all interactions with female anthias and scored the follow–up interactions. We scored whether female anthias had been aggressive or not, and whether the next attack of a blenny was directed at a female anthias or not. Because a time delay could degrade the effectiveness of aggression we ignored the previous interaction if the blenny had spent time in its hole. We thus had for each blenny one probability of switching victim species after being aggressed by female anthias, and one probability of switching victim species without being aggressed by female anthias. These data had been used previously to demonstrate that aggression increases the probability that a blenny switches to a different victim species for its next attack.

Here we asked how important female anthias were for the diet of our blennies and whether or not individual blennies responded with similar switching probability to aggression.

More specifically we calculated for every blenny the percentage of anthias chosen as victims in two ways: as percentage of anthias approached for attack and bitten of all approaches and bites observed in all species (approaches and bites of anthias/all approaches and bites ×100) as well as in a more restrictive way as percentage of bites on anthias in relation to all bites on all species observed (bites of anthias/all bites×100). The effect of punishment was calculated only for the six blennies where we observed at least 50 aggressive responses by female anthias. The measured effect was the percentage of switching after aggression minus the percentage of switching after no aggression. A minimum of 50 data points were selected as criterion on the basis that we wanted to avoid a) low expected values in our chi square test, b) low power of finding a significant difference if it exists due to small sample size, and c) overall high variance in the data (probability of the blenny switching to another victim species) due to small sample size.

### B) Experimental Data in the Laboratory

Experiments were conducted in August 2006 and July 2009 in the Indo Pacific, at the Lizard Island Research Station, Great Barrier Reef, Australia. Subjects were caught in the surrounding reefs and released at the site of capture after the experiments. We only used *Plagiotremus rhinorhynchus* as subjects because they are easier to catch than *P. tapeinosoma*. Individuals were caught by first harassing them to a point where they would hide in their little hole. Then, we placed a hand–net above the hole and sprayed a clove oil solution directly into the hole. The blennies then either fled directly into the hand–net or drifted anaesthetised into the net. Individuals were then put in sealed plastic bags and returned to the station.

The fish were kept individually in opaque aquaria sized 39×30×30 cm. Each aquarium contained a small tube (plastic or bamboo) attached to coral rubble that the fish used as a hiding place. After one day of acclimatisation we started holding forceps with mashed prawn flesh in front of the entrance of the tube 3 times per day. All fish fed after a maximum of six days. In a second step the blennies learned to feed from a small plastic plate (3×2 cm) covered with mashed prawn, again held in front of their tube. Learning took 1 to 5 days of exposure. Once individuals fed off the plate during three consecutive presentations over a day they were ready for the experiments.

**Figure 1:**
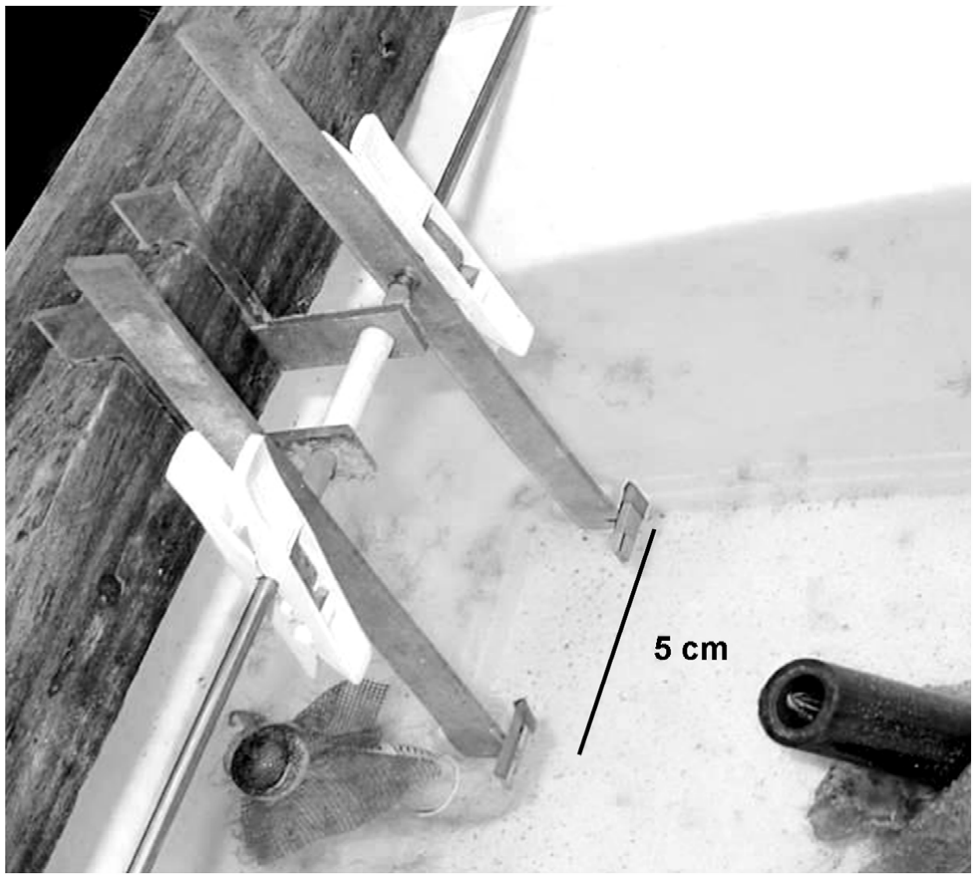
Tank with the construction that was used in the experiments in the laboratory. By pulling a lever towards self until it hit the wooden block the experimenter could cause a standardised punishment action of the attached food–containing plates.

We performed three experiments in the laboratory. Data for the first two experiments were collected during July 2009, testing 8 blennies, all in the same order. Data for the 3rd experiment were collected during August 2006, testing 3 blennies. For all experiments we used Plexiglas plates (Size: 2.0×1.2 cm) that were attached to a lever and could be attached to a wooden construction that allowed a movement of a standardized amplitude when pushed by hand. The two plates were 5.5 cm apart (figure1). The plates varied in their coloration and patterns. For every experiment each blenny was presented a different sub–set of plates and no plate was presented to the same blenny in more than one experiment.

### Experiment 1

#### Choice of target in a setup without punishment: Targets in fixed positions and always in sight

We presented the free swimming blenny two similar looking plates in fixed positions that did not react to the feeding of the blennies. The experiment was terminated after 20 bites or after10 minutes if the fish did not feed any more. The experiment consisted of 2 rounds. In the second round the positions of the two plates were reversed.

### Experiment 2

#### Choice of target in a setup without punishment. Targets in alternating positions and out of sight between trials

We presented two similar looking plates in alternating positions that were removed after each bite (“fleeing”). The feeding round was terminated after maximally 20 bites or after10 minutes if the fish did not feed any more. There was only one round per feeding session.

### Experiment 3

#### Effects of location and behaviour in a randomized setup including punishment

Each blenny was offered three plates in total. Two plates looked very similar to each other and the other one showed a different colour and pattern. All plates reacted in two ways to attacks and showed each reaction with a probability of 50%. They could perform a standardized movement toward the blenny (“chasing/punishment”) or they could be removed from the tank after being attacked (“fleeing”). The three plates were presented pair–wise in all three possible combinations in a counterbalanced way across trials and blennies.

Blennies experienced two sessions per day. In each session, we tried to obtain 18 data points but this was rarely possible because the blennies often stopped foraging before the completion of a session. We stopped experiments after a maximum of 328 bites (N = 243, 248, 328 bites respectively for the three blennies).

For the analyses we tested in each of the blennies:

1. if there was a general side preference. For this we tested if the observed choice of side differed from the expected 50% chance value.
2. if there was a preference for one of the three plates. We tested if the observed choice differed from the expected value. We calculated the expected value by multiplying the total number of observed bites by the percentage of the presence of the given plate. If the blennies had bitten in every trial the expected value would be 33.33% for all the 3 plates. However, as the blennies often stopped feeding within a session or refused to bite in a specific trial, plate presence during trials with a bite varied between 32.1% and 35.4%.
3. if the chasing movement (punishment) led the blennies to switch to the other side for the next bite. To test this we compared the percentage of switching with and without punishment.
4. if the chasing movement (punishment) led the blennies to stop biting of the plates altogether. To test this we compared the percentage of refusals to bite with and without punishment.
5. if the chasing movement (punishment) influenced the choice of plate at the next bite. For this we compared the choices of plates with and without punishment.

For the laboratory experiments X^2^ tests were performed using the free internet service of Preacher [31]. For the analysis of field data we used SPSS 17. For the sign tests we used the table of Darlington [32] provided by the Psychology department of Cornell University.

## Results

### A) Field

#### Choice of victims

**Figure 2:**
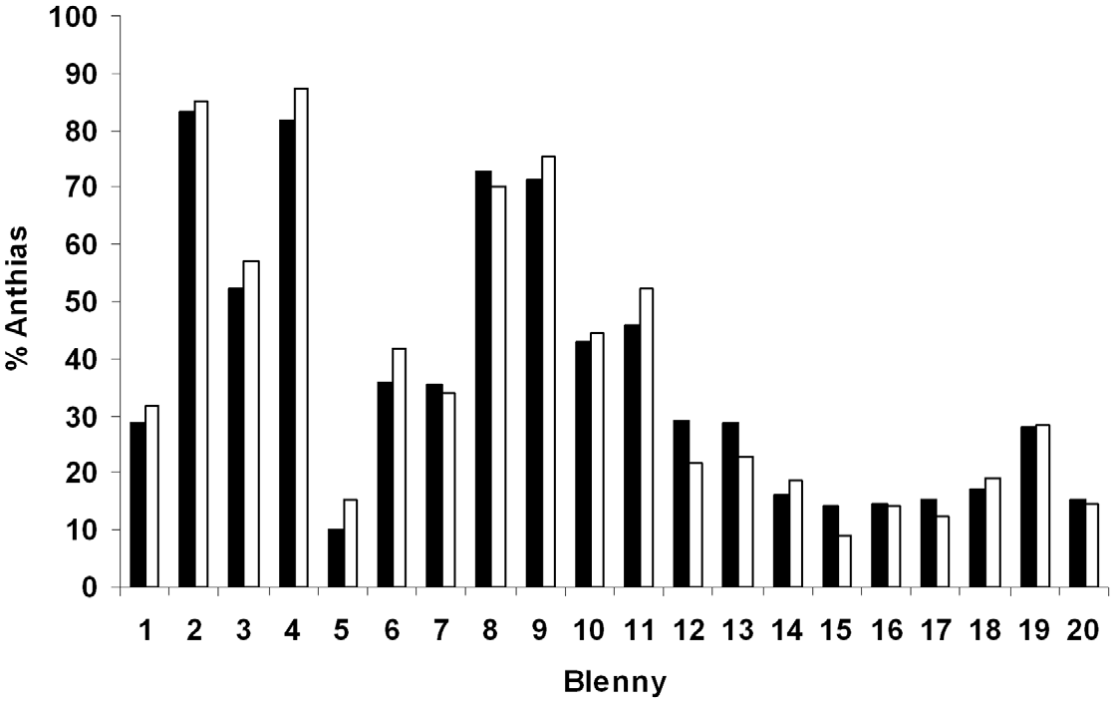
Specialisation on anthias. The proportion of scalefin anthias, *Pseudanthias squamipinnis*, in all the attacks of 20 different blennies observed in the field. Black: Percentage of anthias bitten. White: percentage of anthias approached or bitten.

The percentage of anthias attacked varied significantly between individuals, no matter whether we included or excluded data in which blennies only approached a victim without actually biting it. Values varied between 10% and 83% (approached and bitten) or 9% and 85% (only bitten) respectively (Chi^2^ tests, n = 20 individuals, approached and bitten: N interactions: 3248, Chi^2^ = 503.4, p<0.001, only bitten: N interactions: 2173, Chi^2^ = 392.8, p<0.001, fig. 2).

#### Reaction to punishment/effect of punishment

**Figure 3:**
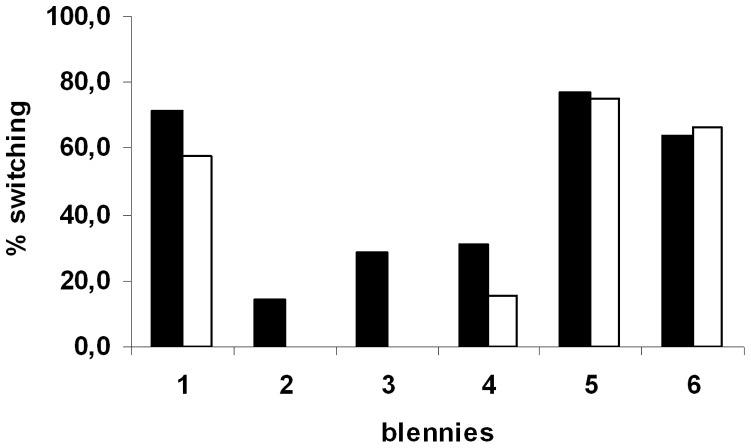
The effect of punishment. Black: the probability of six blennies switching to another species following aggression by a female anthias. White: the percentage of switching to another species without previous aggression. Data shown for the six blennies that were observed being aggressed by female scalefin anthias more than 50 times.

Six blennies were observed being punished more than 50 times by anthias and hence analysed in more detail (see methods). The probability of a blenny switching to another species after being punished varied between 14.1%–71.4%, yielding overall highly significant differences between individuals (Chi^2^ test, n = 560 observations on 6 blennies, Chi^2^ = 227.8, df = 5, p<0.001, fig. 3).

There was no significant correlation between the probability of aggressive reactions (punishment) by anthias and the effectiveness of punishment (measured as the blenny switching to another species following punishment – switching without punishment) though the correlation coefficient was quite positive (Spearman correlation, N = 6, rs = 0.71, p = 0.11). The percentage of aggression was high in all observed groups of anthias, (>66%).

The effect of punishment relative to non−punishment on switching to a different victim species for the next attack was negatively correlated to the probability of switching without punishment (Spearman correlation, N = 6, rs = –0.94, p = 0.005). In other words, the positive effect of punishment was strongest if blennies were generally unlikely to switch to another victim species (blennies 2, 3 and 4 in figure 3), while blennies that were likely to switch even without punishment hardly switched more after being punished (blennies 1 and especially 5 and 6 in figure 3).

### B) Laboratory

#### Experiment 1: Do blennies focus on a location or on a particular victim in the absence of punishment (plate remains in the same spot and in sight between trials)?

**Figure 4:**
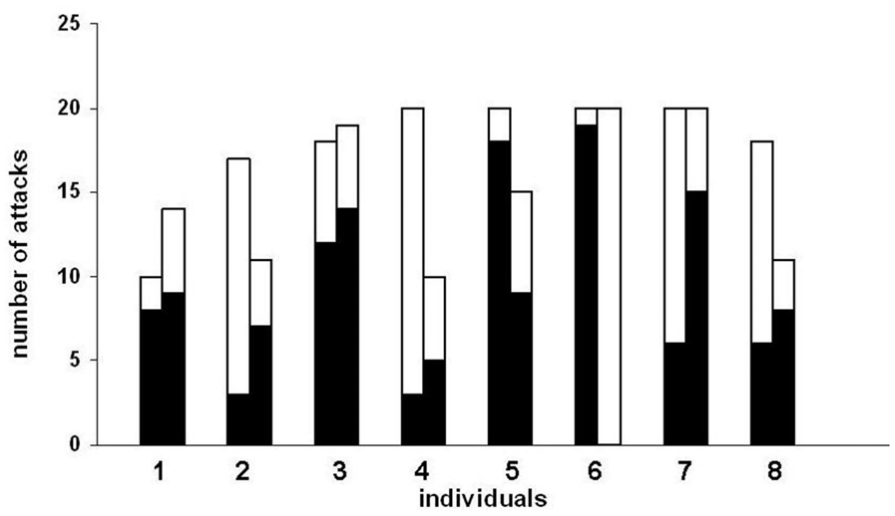
Choice of side in the first experiment. black: left side chosen, white: right side chosen. A similar, asymmetric distribution of black and white indicates a side preference; a dissimilar, asymmetric distribution of black and white indicates a plate preference; a similar, symmetric distribution of black and white indicates random choice.

Combining the data of the two sessions, 3 blennies (3, 4 and 5) showed a significant side preference (Sign tests: n3 = 37, X3 = 26; n4 = 30, X4 = 22; n5 = 35, X5 = 27, all p≤0.02), while blenny 1 only showed a tendency to prefer one side (Sign test: n1 = 24, X1 = 17, p = 0.06). 5 blennies (2, 4, 5, 6 and 7) showed a significant preference for one of the two plates (Sign tests: n2 = 28, X2 = 21; n4 = 30, X4 = 22, n5 = 35, X5 = 24, n6 = 40, X6 = 39, n7 = 40, X7 = 29, all p≤0.04, fig. 4) while blenny 8 only showed a tendency to prefer one plate (Sign test: n8 = 29, X8 = 20, p = 0.06).

#### Experiment 2: Do blennies focus on a location or on a particular victim in the absence of punishment (plate moves and is out of sight between trials)?

**Figure 5:**
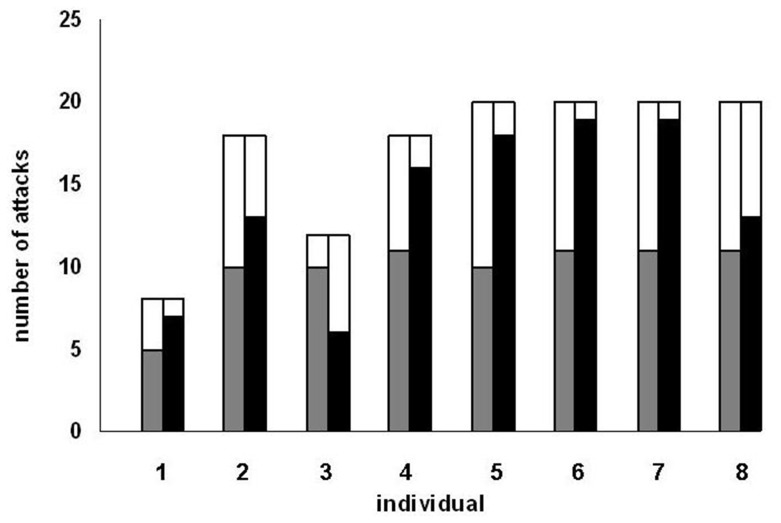
Choice of side and plate in experiment 2. First column: Grey: side chosen more often; white: side chosen less often Second column: Black: plate chosen more often; white: plate chosen less often.

Plates differed in colour and pattern from experiment 1. Only blenny 3 developed a side preference (Sign test: n3 = 12, X3 = 10, p = 0.038, fig. 5). Four blennies (4, 5, 6 and 7) developed a preference for one of the two plates (Sign tests: n4 = 18, X4 = 16; n5 = 20, X5 = 18; n6 = 20, X6 = 19; n7 = 20, X7 = 19, all p≤0.002, fig. 5). Note that the five individuals with significant preferences (3, 4, 5, 6 and 7) kept their preferences from experiment 1 under the new conditions. Three blennies yielded non significant results, where two of them (blennies 1 and 2) showed a tendency to prefer one plate (Sign test: n1 = 8, X1 = 7, p1 = 0.07, n2 = 18, X2 = 13, p2 = 0.09, fig. 5).

#### Experiment 3: Does punishment influence the likelihood of attacking and the choice of target and location? Spontaneous preferences

All three blennies developed a clear side preference (Chi^2^ tests, all Chi^2^ >16, all p<0.001). In addition, all blennies showed a significant preference for specific plates. However, which plate was preferred actually differed between them: One blenny preferred the two look–alike plates over the differently looking plate, one blenny preferred one of the look–alike plates over the other two plates, and the third blenny preferred the different looking plate over the two look–alike plates (Chi^2^ tests, all Chi^2^ >9, all p<0.01).

#### Influence of punishment: Likelihood of attack

Two of the blennies were significantly more likely to stop biting after being punished, one blenny showed a slight tendency in the same direction (Chi^2^ tests, p1 = 0.0035, p2 = 0.036, p3 = 0.091).

#### Choice of target

Punishment influenced the choice of the plate in two of the blennies. One was significantly less likely to bite the same plate after being punished and another showed a tendency to switch to the other plate (Chi^2^ tests, Chi^2^1 = 3.9, p1 = 0.05, Chi^2^2 = 3.05, p2 = 0.081). The latter was also significantly more likely to switch from a look–alike plate to the different looking plate after being punished (Chi^2^ test, Chi^2^ = 9.66, p = 0.002). The switching from the differently looking plate to one of the look–alikes was not influenced by punishment in any of the three blennies (Chi^2^ tests, all Chi^2^ <1.4, all p>0.2).

#### Choice of location

Punishment did not make it more likely that the blennies switched to the other side for the next attack (Chi^2^ tests, all p>0.2).

## Discussion

In previous studies on parasitic blenny–victim interactions we had found evidence for a public good that is maintained through blenny–foraging decisions in two ways [13], [15]. First, the punishment of the parasite is stable because it is self–serving: blennies avoid punishing individuals. Second, a public good emerges because the parasites also are more likely to avoid the whole group after being punished. Individual differences in the foraging decisions of blennies could change the effect of punishment in a way that makes punishment useless and/or does not create a public good. Therefore we were interested to see if there is variation in blenny feeding preferences that could affect both the self–serving aspect of punishment of the parasite and the emergence of a public good in shoals of fish. We found indeed important variation with respect to victim species choice and probability of switching between species with or without punishment. Furthermore, the first two laboratory experiments indicate that individuals are rather consistent with respect to a key initial decision: to focus on a suitable location or to focus on suitable victims. We will first discuss the potential causes of this variation and then discuss the consequences of such variation for the establishment of public goods through self–serving punishment.

### Potential Causes for Individual Differences between Blennies

#### Local victim species composition

One possibility could be that small–scale ecological differences between the territories of the parasites, like differences in the local victim composition, lead to different hunting strategies. Unfortunately, we lost a data sheet containing fish abundances in 2007 and hence cannot properly test this hypothesis. Anthias females were the most abundant victims at all our blocks (this is why the blocks were chosen). However, the abundance of other species was certainly variable at the patches [33]. Different victim species react differently to attacks of blennies. The largest differences exist between species that are resident in the territory of the blenny and species that occupy larger territories and are only briefly visiting the territory of the blennies. Generally, visiting species do not chase the parasites after attacks while resident species regularly show this punishing behaviour [13]. Blennies that occupy territories that are frequently visited by non–punishing fish might preferably bite these victims. But this still has to be confirmed and does not explain the whole range of differences between individual blennies. Other differences between victim species like differences in mobility or size could also lead to preferences in the blennies dependent on local species composition.

#### Specialisation on few victim species

Generally, predators seem to prefer abundant prey types [34] and the abundance of a given species varies between the territories observed. In the field we observed that some blennies specialized on only a few victim species and were not very likely to switch to other species. Such specialisation has been described for many predators and also pollinators [23], [35]–[38]. Feeding efficiency may be improved because the predators can focus on one search image at a time though they may switch between several search images [39].

#### Specialisation on location

In the laboratory we observed that some blennies show a strong preference for the location of attacks. At reef patches where victims show a high probability of aggressive reactions blennies might prefer to attack close to their hiding place to be able to quickly retreat into safety.

#### The potential effects of learning

It is possible that the different specialisations found in our study species are based on individual learning. First, with respect to species composition blennies could learn through operant conditioning which victim species to attack to optimize the energy gain [35], [40]–[42], where initial chance events (success and failure due to victim responses) may lead to specialisation based on differing reinforcement. This would be comparable to optimal foraging rules observed in other predators and pollinators [35], [43], [44]. Second, different decision rules concerning responses to punishment could develop due to the complexity of the blenny’s interspecific ‘social’ world. Due to the great number of victims it may not be optimal for blennies to aim at a perfect knowledge of the system they live in. Instead of having a complete understanding of the reactions of all individual fish in their territory experience might lead to the development of simple rules of thumb like: “after being chased avoid this area for the next attack” or “if you could get a bite without being aggressed keep the victim in view for a possible additional bite”. Such rules of thumb could vary between blennies due to differences in victim species composition and other differences between the territories. In a complex environment, simple rules may maximise the balance between benefits of performing well and the costs of information while individuals forego the possibility to achieve maximal rewards in each specific situation (“bounded rationality” [45]).

#### Personality traits

Much of the literature about individual differences is about personality traits [20], [21], [46]. It is possible that some differences in blenny decision rules might be due to differences in personalities. For example, bold individuals may be less flexible and hence rather unresponsive to victim behaviour while shy individuals incorporate environmental feedback more readily in their foraging decisions. As we have not scored any personality traits in our blennies yet we won’t discuss this possibility any further.

### Potential Consequences of Blenny Foraging Decisions for the Evolution of Punishment and the Emergence of Public Goods

The victim species we studied in detail, *Pseudanthias squamipinnis*, seems to be rather inflexible in its response to blenny–attacks. Apart from the slightly negative correlation between local abundance and probability of punishment we found in our previous study [13], there is apparently not much variation in the probability of aggressive responses, which was always above 60%. Thus, we can focus on the question how variation in blenny strategies may affect a) the self–serving effect of punishment, and b) the emergence of public goods as a side effect.

There are three main scenarios illustrating the importance of variation in blenny feeding strategies:

- In locations where the blenny is very likely to switch anyway between species no matter if it was punished before or not, punishment is not functional. If switching was the blennies’ standard strategy, then individual victims would be under selection not to punish. There would be no public good but also no competition between look–alikes because the behaviour of the blenny is not influenced by the behaviour of the victims.
- If the blenny switches to another individual if punished but not to another species then punishment pays for the individual. But this blenny strategy will lead to competition between look–alikes rather than to a public good, because for conspecifics punishment increases rather than decreases the risk of being attacked.
- Only if punishment causes the blenny to switch to another species does punishment pay for the individual while providing a public good by decreasing the risk of future attacks for both the individual and its conspecifics.

Here we make some predictions how variation in a blenny’s preference for certain locations and a focus on individual recognition and book–keeping of victim responses may lead to punishment being self–serving or not, and providing a public good or not. These predictions are amenable for future testing.

First, blennies with a strong preference for a location are highly likely to cause repeated interactions within short time periods. This is the situation where punishment may be effective if the victim lives in the core area. Outside the core area interactions will be infrequent and punishment won’t pay because the blenny is very likely to switch to another victim anyway. Alternatively, a blenny roves within its territory and hence is likely to switch automatically between victims because of the roving. In such circumstances punishment does not provide benefits to the punisher. Second, blennies that mainly pay attention to individual identity select for punishment but such punishment could cause either increased competition or a public good. An increase in competition would be more likely if the blennies remember an experience with a specific individual rather than with a species. If blennies mainly avoid location in response to punishment then they may shift microhabitat, which should increase the likelihood that they switch to another species [26], [27], and hence punishment would pay for the individual and create a public good. The predictions are summarised in Table 1.

**Table 1:** Predictions how aspects of blenny foraging rules affect the efficiency of punishment and the emergence of a public good in shoaling victim species *with a spatial structure*.

	Aspects of blenny foraging rules		
	Roving	Preference for location/species	Preference for non-punishing individuals
**Blenny reaction to punishment:**	none; switches anyway between individual/species	switches to another individual/species	switches to another individual/species
**Punishment provides:**	no benefit	benefit for individual	benefit for individual
**Without punishment**	no difference	blenny is likely to return to same location	blenny is likely to return to same individual
**Effect of punishment** **on con-specifics**	no effect	decreases risk	increases risk
**Public good**	no public good	public good	no public good

### On the Stability of Punishment and Public Goods

Our field observations and laboratory experiments demonstrate that individual blennies are indeed variable with respect to the importance of location and victim identity for their foraging decisions. Thus, the major open question at this stage is whether the observed variation in blenny decision rules, which sometimes renders punishment ineffective or merely self–serving, and which may affect look–alikes in either positive or negative ways, helps overall to stabilise the emergence of a public good due to self–serving punishment [15]. Several models of cooperation yield stable cooperation because variation is maintained by ontogenetic effects (‘phenotypic defectors’ in [47]) or mutation rates [20]. Thus, the observed variation may indeed help to stabilise contributions to the public good in our system. On the other hand, one has to explain why anthias seem to be rather inflexible in their decision rules and always show high levels of aggressive responses. Again, a game theoretic exploration of the observations may help to generate more specific predictions amenable for future testing.
